# Preparation of Trichloride and Tetrachloride of Molybdenum

**DOI:** 10.6028/jres.063A.013

**Published:** 1959-10-01

**Authors:** Dwight E. Couch, Abner Brenner

## Abstract

Molybdenum trichloride was successfully prepared in quantity by the reduction of molybdenum pentachloride with hydrogen. The most satisfactory yields were obtained with a 4 to 5 mole excess of hydrogen at pressures of 100 psi or higher and at a temperature of 125°C.

Molybdenum tetrachloride was prepared by direct reaction of molybdenum trichloride with molybdenum pentachloride in a sealed tube or steel bomb maintained at 250° C. X-ray patterns of the various chlorides were obtained.

## 1. Introduction

Blomstrand [1]^1^ prepared low-valence molybdenum halides by passing hydrogen over the pentachloride in a hot tube. The process has been modified many times. Liechti and Kempe [2] used a similar procedure and claimed to have prepared molybdenum tetrachloride by condensing the vapors resulting from the thermal decomposition of the trichloride. Michael and Murphy [3] prepared molybdenum tetrachloride and pentachloride by reacting the di- and trioxide with carbon tetrachloride at 250° C. Linder, Haller, and Helwig [4] prepared molybdenum dichloride by the reaction of molybdenum with phosgene at about 600° C. However, Biltz and Fendius [5] claimed that the tetrachloride had never been prepared. They prepared the dichloride by disproportionation of the trichloride. Wardlaw and Webb [6] studied molybdenum pentachloride in organic solvents and found that it reacted with dry pryidine to form pyridine-molybdenum tetrachloride, but they were not able to remove the pyridine from this salt completely. Forland [7] obtained a patent for the preparation of molybdenum chlorides by the direct chlorination of molybdenum disulfide.

Hellriegel [8] prepared molybdenum di- and trichloride from molybdenum and molybdenum pentachloride. Senderoff and Brenner [9] tried several reported methods in an effort to prepare the lower molybdenum chlorides. They found that the direct reduction of pentachloride by hydrogen [1] was very slow and gave only a small yield of molybdenum trichloride, and also reported the reaction between molybdenum and phosgene to be unsatisfactory. They prepared the trichloride by a procedure similar to that of Hellriegel [8] and also prepared the dichloride from the trichloride. Senderoff and Labrie [10] attempted to prepare molybdenum trichloride by reduction of the pentachloride with hydrogen, aluminum, and sodium, but failed to develop a suitable procedure. They prepared molybdenum tetrachloride containing organic material by heating molybdenum pentachloride with cetane or paraffin and obtained the trichloride by heating this product.

Since the tetrachloride and trichloride were required in moderate quantities for electrolytic studies, attention was directed toward the development of more convenient methods of making these compounds. As molybdenum pentachloride is now commercially available, it was used as the starting material. The trichloride was readily prepared by hydrogenation of the pentachloride under pressure at about 125° C. The reaction was unusual in that it proceeded to completion only with solid pentachloride. The tetrachloride was prepared by heating the trichloride and the pentachloride together in a sealed tube.

## 2. Preparation of Molybdenum Trichloride

Ordinary high pressure equipment was used for all hydrogen reductions discussed in this paper. It consisted of a stainless steel bomb equipped with glass liner. The bomb head was equipped with a pressure gauge and a three-way valve for evacuating the bomb and for subsequently adding the hydrogen. The bomb was heated with an external resistance furnace. All chemicals were handled in an inert atmosphere to prevent contamination of the molybdenum compounds with moisture and oxygen. After assembly, the bomb was evacuated, flushed once with hydrogen, refilled with hydrogen, and heated.

The determination of the yield of molybdenum trichloride was accomplished by mixing the reaction product with 1:1 hydrochloric acid-water solution in which the trichloride is insoluble, then filtering and drying the residue in a vacuum desiccator. This procedure gave the yield to within 2 percent, an accuracy sufficient for this work.

A few preliminary reductions of the pentachloride were made with an excess of hydrogen at a temperature of 200° C and a pressure of 5 to 10 atm (see table 1). An inspection of the reaction product indicated that only the upper surface of the pentachloride had reacted and a solid crust of molybdenum trichloride was formed on top of the liquid pentachloride. The reaction was slow because of the low rate of diffusion of hydrogen through this crust of molybdenum trichloride. Experiments 1 and 2, table 1, show that the procedures reported in the literature are not satisfactory for preparing molybdenum trichloride.

The reduction of pentachloride to trichloride was 98 percent complete after 16 hr at 125° G with a 5 to 1 mole ratio of hydrogen to pentachloride. The temperatures and pressures were not critical as long as there was an appreciable excess of hydrogen and the temperature was between 120° C and the melting point of molybdenum pentachloride, 190° C. Results of successive treatments with fresh hydrogen indicated that once the molybdenum trichloride had formed it was stable at the conditions used, 125° C, and was not reduced to the dichloride.

The last traces of molybdenum pentachloride and hydrogen chloride were removed from the trichloride by heating it in an inert atmosphere or in a vacuum after the reduction. Generally, this was accomplished by heating to 250° C under vacuum for about 15 min. Chemical analysis of a typical preparation gave 46 percent of molybdenum and 52 percent of chlorine. (Theoretical: 47.5 percent of molybdenum and 52.5 percent of chlorine.) The trichloride prepared in this way was practically insoluble in aqueous hydrochloric acid. However, if it was allowed to stand in contact with water for several hours, some oxidation took place as shown by the formation of a blue molybdenum solution. It also formed oxychlorides if allowed to remain in contact with moist air for several weeks. The trichloride was insoluble in dimethyl formamide, acetone, pyridine, acetic anhydride, ethyl alcohol, ethyl ether, and dimethyl cyanamide, but was soluble in ethyl pyridinium bromide at 110° C.

The molybdenum trichloride prepared by hydrogenation involved a solid phase reaction, and nonvolatile impurities remained in the product. It was desirable to purify molybdenum trichloride by sublimation to compare the properties of the solid-phase reaction product with that deposited from the gaseous phase. However, molybdenum trichloride decomposes on heating according to the reaction:

$$12\;{\text{MoCl}}_{3}\overset{\text{heat}}{\to }{\text{Mo}}_{6}{\text{Cl}}_{12}(s)+6\;{\text{MoCl}}_{4}(g)$$

and on cooling,

$$2\;{\text{MoCl}}_{4}(g)\overset{\text{cool}}{\to }{\text{MoCl}}_{3}(s)+{\text{MoCl}}_{5}(s)[11]$$

Three sections of glass tubing connected by 24/40 standard taper joints were used for preparing the molybdenum trichloride. One end, fitted with an inert gas inlet tube, was charged with molybdenum trichloride. The tube was then heated to about 500° C while a slow stream of dry helium passed through it. The molybdenum trichloride decomposed to yield a bright yellow residue of dichloride and a brown vapor. When allowed to cool, this vapor disproportionated to form a dark solid which was composed of trichloride and pentachloride. After the molybdenum trichloride was decomposed, the center section of the tube was heated to 300° C and the molybdenum pentachloride distilled into the third section. The apparatus was then placed in an inert atmosphere chamber and disassembled. In this way it was possible to isolate all three of the reaction products, molybdenum di-, tri-, and pentachlorides.

Excessive heating of the molybdenum dichloride did not contaminate the trichloride in the experiment since the dichloride also decomposes to the tetrachloride when heated above 700° C. In fact, both molybdenum trichloride and pentachloride can be prepared by heating the dichloride according to the following equations [11]:

$$\begin{array}{l} 2\;{\text{MoCl}}_{2}(s)\overset{700{}^{\circ}}{\to }\text{Mo}(s)+{\text{MoCl}}_{4}(g)\\ 2\;{\text{MoCl}}_{4}(g)\overset{\text{cool}}{\to }{\text{MoCl}}_{3}(s)+{\text{MoCl}}_{5}(s) \end{array}$$

## 3. Preparation of Molybdenum Tetrachloride

The preparation of tetrachloride by the disproportionation of trichloride, as reported by Liechti and Kempe [2], was attempted. Chemical analysis of the sublimate from this reaction indicated that the product was not molybdenum tetrachloride; approximately one-half of it was insoluble in water and was found to be molybdenum trichloride. Thus, the material they prepared was apparently an equimolar mixture of trichloride and pentachloride. Molybdenum tetrachloride prepared by the authors according to the procedure described by Senderoff and Labrie [10] was assayed for molybdenum trichloride. The product prepared with cetane at 165° C contained 40 percent of material insoluble in 1:1 HCl, assumed to be trichloride; that prepared with paraffin at 145° C contained only G percent of insoluble material. Both of these preparations contained organic contaminants. The tetrachloride prepared according to the procedure described by Michael and Murphy [3] contained no detectable trichloride, was completely soluble in 1:1 hydrochloric acid, and was slightly soluble in carbon tetrachloride.

An attempt was made to prepare tetrachloride by the controlled reduction of the pentachloride with hydrogen. With smaller amounts of hydrogen than are theoretically necessary to produce molybdenum tetrachloride (experiment 4, table 1), the product contained 4 percent trichloride, 36 percent of tetrachloride, and 60 percent of pentachloride. A second reduction (experiment 7, table 1) using the stoichiometric amounts of hydrogen needed to produce tetrachloride gave a mixture of halides containing 8 percent of trichloride, 74 percent of tetrachloride, and 18 percent of pentachloride. It was evident from these reactions that the molybdenum tetrachloride prepared in this way would always be contaminated with both trichloride and pentachloride.

Molybdenum tetrachloride was successfully prepared by direct reaction of pentachloride and trichloride in a sealed tube. This reaction was carried out by sealing about 5 g of the finely ground and mixed halides in a 14-mm ID gage-glass tube filled with helium or argon. All operations were performed in an inert atmosphere. The completeness of the reaction was determined by an assay for molybdenum trichloride based on its insolubility in 1:1 hydrochloric acid. Molybdenum tetrachloride and pentachloride are soluble. Table 2 shows the results of several different preparations. Experiments number 3 and 4, Table 2, indicate that the reaction was more nearly complete at 250° C than at 400° C.

The high yield of molybdenum tetrachloride is not entirely in agreement with the calculated data presented by Quill [11], according to which the tetrachloride is not stable when cooled from temperatures above 125° C. It is probable that the pressure in the tube and the excess of pentachloride shift the equilibrium in a favorable direction.

Since an excess of molybdenum pentachloride was required to convert all the trichloride to tetrachloride in a reasonable time, it was necessary to find a method of separating the pentachloride from the tetrachloride. A large number of anhydrous organic solvents were tested, but none was satisfactory. In fact, no solvent was found which would dissolve either halide in appreciable quantities without reacting with it.^2^

The products from the reaction of molybdenum pentachloride with molybdenum trichloride were subjected to vacuum sublimation at 120°C. The results showed that the pentachloride could be distilled out without causing any thermal decomposition of the tetrachloride. If any disproportionation was taking place during these sublimations, it would have been evident from the formation of the trichloride. However, an assay of both the sublimate, which was the pentachloride, and the residue, which was the tetracliloride, showed only traces of trichloriode. A chemical analysis of the residue from the sublimation showed that it contained 38 percent of molybdenum and 59 percent of chlorine. (Theoretical: 40.3 percent of molybdenum and 59.7 percent of chlorine.)

## 4. X-Ray Studies

Table 3 shows the *d* spacings and relative intensities of the molybdenum penta-, tetra-, tri-, and dichlorides. These data have not been reported previously. The molybdenum tetrachloride prepared by reacting the trichloride with the pentachloride and that prepared in accordance with Michael and Murphy [3] and Senderoff and Labrie [10] had similar patterns. Diagrams of the molybdenum trichloride prepared by hydrogen reduction and by thermal decomposition were identical. *_t_*Furthermore, after the trichloride was washed with dilute hydrochloric acid and then dried in a vacuum desiccator, it still gave the same pattern showing that it was not affected by short-time treatments with dilute hydrchloric acid, but if allowed to- stand in contact with moist air the pattern of trichloride was changed. The X-ray pattern of molybdenum dichloride which had been washed with dilute nitric acid or allowed to stand for a long period of time and dried, differed from that of the pure dichloride. This was rather surprising since the compound apparently did not take up moisture and showed no visible signs of reacting with the nitric acid.

## Figures and Tables

**Table 1:** Preparation of molybdenum trichloride by reduction with hydrogen

Exp.	Temp	Time	H_2_ pressure	Wt MoCl_5_ used	Wt loss	Mole ratio H_2_:MoCl_5_	MoCl_3_ in product
							
	° *C*	*hr*	*psi*	*g*	*g*		%
1	125 to 160	4	14.7	50	……………	…………………………	None
2	250 to 275	12	14.7	200	……………	…………………………	5
3	200	65	1375	100	……………	25:1	79
4	145	1	90	211	13.5	0.4:1	4
5	135	0.5	1100	50	9.6	36:1	80
6	125	0.5	100	50	10.7	4:1	80
7	125	16	55	100	12	0.5:1	8
8	125	16	150	50	12	5:1	98
9	125	18	450	50	12	16:1	98
10	125	12	450	50	11	18:1	97
11	85	16	1600	50	0.5	58:1	None
12	25	16	1750	100	None	31:1	None

**Table 2:** Preparation of molybdenum tetrachloride by direc reaction of the trichloride with pentachloride

Exp.	Mole ratio MoCl_5_/MoCl_3_	Temp	Time	MoCl_3_ in product
				
		° *C*	*hr*	%
1	pure MoCl_3_	250	150	99
2	2.5:1	250	70	trace
3	2:1	400	20	9
4	2:1	250	20	3
5	1.5:1	150	150	14
6	1.5:1	250	150	2
7	1.2:1	250	70	5
8^a^	1.2:1	250	150	3
9	1.2:1	250	500	4
10	1:1	250	40	10
11	1:1	250	70	7
12	1:1	250	150	4

a 100-g batch in a steel bomb.

**Table 3:** X-ray diffraction data on various molybdenum halides showing d spacing and relative intensities, taken with copper radiation, wavelength 1.5405 A (by Howard E. Swanson)

MoCl_2_		MoCl_3_		MoCl_4_^a^		MoCl_5_		MoCl_5_ (Continued)	
*d*	*I*	*d*	*I*	*d*	*I*	*d*	*I*	*d*	*I*
									
*A*		*A*		*A*		*A*		*A*	
7.02	100	5.99	100	6.85	8	8.68	5	2.533	5
6.39	12	5.17	8	6.52	6	6.87	5	2.481	10
5.64	26	4.94	7	5.85	100	5.76	55	2.406	5
3.46	8	4.78	9	5.36	8	5.68	100	2.354	5
2.90	4	3.60	5	5.26	8	5.30	40	2.347	15
2.84	6	3.05	2	5.16	5	5.24	55	2.297	5
2.78	5	2.86	4	4.79	18	5.12	30	2.273	5
2.77	5	2.689	10	4.42	6	5.07	55	2.226	15
2.61	12	2.599	14	4.14	6	4.83	5	2.195	10
2.55	6	2.438	5	3.14	3	4.67	10	2.137	5
2.52	8	2.167	2	3.11	6	4.54	10	2.092	10
2.37	9	2.104	5	3.04	3	4.33	20	2.080	5
2.34	9	2.078	7	2.925	5	4.29	15	2.054	10
		1.998	4	2.696	80	4.24	20	1.975	30
2.33	5	1.909	5	2.647	9	4.20	30	1.969	10
2.31	8	1.889	3	2.564	3	4.17	10	1.866	10
		1.726	13	2.444	2	4.05	10	1.857	5
2.20	13	1.661	4	2.106	54	3.94	5	1.768	5
2.09	10	1.631	5	1.962	5	3.74	5	1.758	10
2.08	8	1.498	4	1.944	4	3.68	25	1.743	30
2.045	14	1.395	*2*	1.752	22	3.59	<5	1.688	5
2.014	9	1.289	2	1.678	10	3.48	20	1.668	10
1.997	9	1.198	1	1.642	18	3.36	15	1.642	5
1.991	10	…………	…………	1.636	2	3.22	10	…………	…………
1.946	10	…………	…………	1.468	8	3.11	5	…………	…………
1.930	9	…………	…………	1.456	7	3.07	10	…………	…………
1.911	9	…………	…………	1.346	4	2.968	5	…………	…………
1.903	8	…………	…………	1.196	3	2.895	20	…………	…………
1.779	10	…………	…………	1.128	4	2.877	20	…………	…………
1.757	7	…………	…………	1.120	3	2.810	5	…………	…………
1.753	3	…………	…………	…………	…………	2.756	35	…………	…………
1.738	5	…………	…………	…………	…………	2.730	25	…………	…………
1.736	4	…………	…………	…………	…………	2.684	25	…………	…………
1.678	1	…………	…………	…………	…………	2.633	60	…………	…………
1.470	6	…………	…………	…………	…………	2.592	30	…………	…………

a Average of two patterns of MoCl_4_, one prepared from MoCl_3_+MoCl_5_ and the other prepared from MoO_2_+CCl_4_.

## Footnotes

2 Roger J. Labrie of the NBS has recently found that titanium tetrachloride will dissolve molybdenum pentachloride but not molybdenum tetrachloride.
